# Law enforcement and syringe services program implementation in rural counties in Kentucky: a qualitative exploration

**DOI:** 10.1186/s12954-022-00684-8

**Published:** 2022-09-30

**Authors:** Sean T. Allen, Sarah Danforth, Suzanne M. Grieb, Jennifer L. Glick, Samantha J. Harris, Catherine Tomko, Susan G. Sherman

**Affiliations:** 1 Department of Health, Behavior and Society, Johns Hopkins Bloomberg School of Public Health, Johns Hopkins University, 624 N. Broadway, Hampton House 184, Baltimore, MD 21205 USA; 2 Department of Pediatrics, Center for Child and Community Health Research, Johns Hopkins School of Medicine, Baltimore, MD 21224 USA; 3 Department of Health Policy and Management, Johns Hopkins Bloomberg School of Public Health, 624 N. Broadway, Baltimore, MD 21205 USA

**Keywords:** Syringe services program, Rural, Injection drug use, HIV, People who inject drugs, Drug policy

## Abstract

**Background:**

Existing research in urban areas has documented a multitude of ways in which law enforcement may affect risks for bloodborne infectious disease acquisition among people who inject drugs (PWID), such as via syringe confiscation and engaging in practices that deter persons from accessing syringe services programs (SSPs). However, limited work has been conducted to explore how law enforcement may impact SSP implementation and operations in rural counties in the United States. This creates a significant gap in the HIV prevention literature given the volume of non-urban counties in the United States that are vulnerable to injection drug use-associated morbidity and mortality.

**Objective:**

This study explores the influence of law enforcement during processes to acquire approvals for SSP implementation and subsequent program operations in rural Kentucky counties.

**Methods:**

From August 2020 to October 2020, we conducted eighteen in-depth qualitative interviews among persons involved with SSP implementation in rural counties in Kentucky (USA). Interviews explored the factors that served as barriers and facilitators to SSP implementation and operations, including the role of law enforcement.

**Results:**

Participants described scenarios in which rural law enforcement advocated for SSP implementation; however, they also reported police opposing rural SSP implementation and engaging in adverse behaviors (e.g., targeting SSP clients) that may jeopardize the public health of PWID. Participants reported that efforts to educate rural law enforcement about SSPs were particularly impactful when they discussed how SSP implementation may prevent needlestick injuries.

**Conclusions:**

The results of this study suggest that there are multiple ways in which rural SSP implementation and subsequent operations in rural Kentucky counties are affected by law enforcement. Future work is needed to explore how to expeditiously engage rural law enforcement, and communities more broadly, about SSPs, their benefits, and public health necessity.

## Background

Implementing syringe services programs (SSPs) is an effective strategy communities may employ to reduce community-level risks for injection drug use-associated infectious disease outbreaks among people who inject drugs (PWID) [1–6]. These programs may offer numerous health and social services including: access to sterile injection equipment (e.g., syringes, cookers), substance use disorder treatment referrals, testing for HIV and sexually transmitted infections (STIs), vaccinations, and overdose prevention resources [1, 3, 4, 7, 8]. SSPs have been studied for more than three decades, leading to extensive evidence that their implementation carries substantial public health benefits and does not encourage drug use or lead to increased syringe litter [9–12]. While empirical evidence demonstrates the utility of SSPs, their implementation may be obstructed by a myriad of factors, including drug paraphernalia laws and law enforcement practices [13–17].

Many studies have documented that policing practices are associated with a range of adverse health behaviors and consequences among PWID [15, 18–21]. For example, police confiscation of sterile injection equipment may lead to increased syringe sharing and exacerbate risks for HIV transmission [20, 21]. Other research has shown that intensified police presence may lead PWID to rush injections or inject in unsafe environments, thus discouraging safer injection practices [19]. In addition, police action and threats of police action are associated with decreased SSP utilization, which may in turn increase risks for overdose and infectious disease acquisition [22]. A study in Baltimore, Maryland also found that PWID traveling to and from SSP locations had increased risk of arrest and having legally obtained syringes destroyed by police [14].

Existing literature that examines how law enforcement practices affect the public health of PWID and SSP implementation primarily reflect studies conducted in urban areas, creating a significant gap in how we understand these relationships in rural contexts. Unique contextual factors for rural communities (e.g., diminished access to evidence-based drug treatment, greater prescription opioid misuse, greater levels of chronic pain) underscore the importance of understanding SSP implementation in non-urban areas [23]. This deficit in the literature has grown in importance in recent years given that many rural states, like Kentucky and West Virginia, launched SSPs following the 2015 injection drug use-associated HIV outbreak in rural Scott County, Indiana. Subsequent analyses identified 220 predominantly rural counties that were vulnerable to similar outbreaks [24].

Enhanced understanding of how law enforcement may affect the implementation and subsequent operations of SSPs in rural contexts and, by extension, the public health of PWID populations, is vital to both successfully launching programs and sustaining their operations. In 2015, the Kentucky legislature authorized the implementation of SSPs provided that three entities approved: the Board of Health at a local health department, county fiscal courts, and city councils [25]. This study explores the influence of law enforcement during processes to acquire approvals for SSP implementation and subsequent program operations in rural Kentucky counties.

## Methods

This analysis was part of a larger study that aimed to understand the barriers and facilitators to SSP implementation in rural Kentucky counties. In-depth, semi-structured interviews with persons (n = 18) who were involved with SSP implementation in rural Kentucky counties were conducted from August-October 2020. Involvement in SSP implementation was broadly defined as persons who advocated for SSP implementation, operated a program, or oversaw SSP activities. Potential participants were identified via searches of publicly available literature related to SSPs, such as media reports and governmental reports. In addition, specific individuals who were described during interviews as having played a role during SSP implementation were considered for potential recruitment. Eligibility criteria included persons being at least 18 years of age and having played a role during SSP implementation in at least one rural county in Kentucky.

Potential participants were first contacted via email, informed about the study, and asked if they would be willing to participate. Interested persons were then scheduled for an interview via Zoom or phone. The first author conducted all of the interviews. All persons were given the opportunity to ask questions about the study prior to beginning the interview, and participants provided oral consent. Interviews lasted approximately 45 min and were audio recorded with participants’ permission. Each participant was offered a $25 gift card as an incentive. The Institutional Review Board at the Johns Hopkins Bloomberg School of Public Health approved this study.

### Interview guide

Given the breadth of factors that may affect SSP implementation, the interview guide for the larger study (which broadly aimed to explore the barriers and facilitators to SSP implementation in rural Kentucky counties) was informed by the Consolidated Framework for Implementation Research (CFIR) and Kingdon’s multiple streams model of policy change [26, 27]. The CFIR offers a systematic way to explore program implementation and includes a robust set of constructs related to implementation processes [26]. During the interviews, participants were asked to describe the process of acquiring approvals for SSP implementation and operating a program; subsequent questions reflected constructs within the CFIR. Kingdon’s multiple streams model of policy change suggests that three “streams” (i.e., the problem, policy, and politics streams) must align for policy change to take place [27]. We incorporated elements from this model into the interview guide for the larger study given that SSP implementation in Kentucky may require an array of policy-level changes (e.g., decriminalizing syringe possession, acquiring multiple approvals prior to program launch) and involve diverse constituency groups [27]. The preliminary interview guide was piloted with our study team and refined to better explore SSP implementation processes. Throughout the interviews, participants discussed law enforcement relative to several questions, such as: “Can you tell me about the influential people in your community who advocated for or against SSP implementation?”, “Why were [constituency groups] for or against SSP implementation?”, “What role did law enforcement play during SSP implementation?”, and “How did these individuals demonstrate their support or opposition to SSP implementation?”.

### Analysis

Interviews were professionally transcribed verbatim, and identifying information was removed from resulting transcripts. A preliminary coding scheme was developed using a priori codes that reflected the goals of the larger study and focal areas of the CFIR and Kingdon’s multiple streams model of policy change. In conjunction with the PI, two qualitative coders refined the coding framework. Specifically, team members read three transcripts and identified emergent themes to create a draft codebook of a priori and inductive codes. Three transcripts were subsequently independently coded by the three team members. The initial application of codes was compared among team members and code definitions were refined as needed. This process was repeated on three more transcripts to generate the final coding framework. Team members then applied the codes systematically to transcripts in MAXQDA software. Each transcript was double coded. The research team met weekly during the coding process to discuss findings. During these meetings, the PI ensured intercoder agreement via monitoring coding comparability and resolving discrepancies.

For the present analysis, we focused on examining coded text pertaining to law enforcement. Law enforcement-related text was defined broadly to include any mentions of law enforcement, such as: police officers interacting with SSP clients and staff, law enforcement advocating for or against program implementation, and other policing behaviors that may have influenced SSP implementation or the public health of PWID. Quotes were then categorized based on how participants described the role of law enforcement relative to SSP implementation and the public health of PWID. Direct quotes are used to demonstrate our findings. All of our participants discussed law enforcement during their interviews. Given the rural nature of our study context, we removed all information about where participants lived or worked to protect their anonymity. We do, however, provide an overview of our participants’ demographics, backgrounds, and roles during SSP implementation.

## Results

Eighteen in-depth interviews were conducted (10 women, 8 men). Among our participants, the majority were White (88.9%). Many participants had been personally affected by the opioid overdose crisis (e.g., friends or family used drugs, knew someone who had overdosed). Participants’ professional roles were diverse, including health department directors, SSP operators, program directors, healthcare providers, and members of the public health workforce. Participants reported a variety of ways in which they were involved in SSP implementation, including via advocacy activities, coalition building, and operating programs. Broadly, participants described that the law enforcement community had a heterogeneous response to SSP implementation across rural counties in Kentucky. A participant succinctly explained the diversity of responses to SSP implementation among law enforcement across rural Kentucky counties by stating:

Some law enforcement police stations are totally behind the syringe services programs. And others are accepting of it, but not wholeheartedly. And then on the other extreme, we have one sheriff who will absolutely refuse to have a syringe services program in his county and he's actually been known to sit at the county line waiting for his county residents to return from the neighboring county's syringe services programs so he can pull them over and take all of their syringes.

### Influence of law enforcement on rural SSPs

Many participants described law enforcement as having a strong influence over rural SSP implementation. As succinctly stated by a participant, “If that health department does not have their local law enforcement onboard with opening a syringe service program or running a syringe service program it will never happen…”. Similarly, another participant explained that having the support of law enforcement can be a strong determinant over whether initial approvals for SSP implementation are granted:

When you've got law enforcement behind you and the Sheriff is actually in the county fiscal court meeting and is standing up saying, ‘I've heard that Sheriff So-and-So from this county is seeing benefits from this program. I think we should do this,’ then that's a very strong voice in that community.

In addition, participants explained that while members of the law enforcement community may not sit on the boards or councils required to approve SSP implementation, they were still influential given their longstanding relationships with policymakers in rural communities. A participant explained this by stating:

…so they had the chief of police, who was against it (SSP implementation) from the beginning, and he does not sit on the city council, but obviously he has some pull there. He's been against it from the beginning…

Another participant explained that engaging law enforcement in rural communities in discussions about SSPs and working to address their concerns may be particularly impactful given that they may be in positions of influence with local elected officials and governing bodies, “… that’s who (law enforcement) a lot of government officials are going to look to and say, ‘What do you think about this?’”.

Participants also elaborated that having current or former members of law enforcement advocate for rural SSP implementation was key to cultivating support among active law enforcement personnel who may not have initially supported program implementation. In essence, participants explained that rural SSPs advocates who were involved in law enforcement were more effective messengers to active law enforcement officers about the need for program implementation than persons from public health backgrounds. A participant described the ability of former law enforcement officials to persuade active members of law enforcement to support SSP implementation by stating:

I can remember a meeting we had in [County Name] very early on in early 2016, and they ended up bringing (a member of) law enforcement into this meeting who was adamantly opposed to it (SSPs) when he arrived and, after he got the great [name of former law enforcement official] attack and the comments from others around the table, he was a firm believer in it (SSP implementation). But it went to show you the role that law enforcement has in various communities and how they're tied politically to various things.

### Attitudes and beliefs about SSPs

Participants frequently described scenarios in which members of the law enforcement community in rural areas questioned the purpose of SSPs providing sterile injection equipment to PWID. For example, a participant explained that some members of law enforcement did not initially know the public health utility of providing sterile injection equipment to PWID and that addressing the underlying attitudes and beliefs of law enforcement officers required education:

There’s kind of a visceral response that [law enforcement] has, ‘What? We’re going to give needles to people who use drugs? Why would we do that?’ And unless you take the time to think and learn about it, you stick with that visceral response, ‘Why would we do that?’ I’m a big believer that change happens at the local level…

Many participants also explained that members of law enforcement viewed SSP implementation in rural communities as something that would enable drug use among PWID. A participant, for instance, stated that some law enforcement officials viewed SSPs negatively and felt that public health personnel who worked at SSPs were enabling drug use: “Law enforcement still has the opinion that needle exchange is not a good thing, we're just enabling…” Another participant similarly shared, “You have real traditional jailers and law enforcement and folks like that who really see our work (SSP implementation) as enabling people.”

### Adverse police behaviors

Several participants described rural law enforcement engaging in behaviors that adversely affected SSP operations. For example, a participant reported that police would park near a SSP and target their clients for drug possession:

…they'll have police literally standing outside, parked around the corner. As soon as [SSP clients] walk out with their supplies, they get arrested and go to jail, and not because of the needles but because most likely they have drugs in their vehicle as well.

Participants further elaborated that law enforcement would confiscate sterile injection equipment and that this behavior not only affected the public health of SSP clients, but also the syringe return rate at the program. As explained by a participant, “You’re never going to get a hundred percent [syringe return rate]. It’s just not going to happen. It isn’t. And, like I said, people get their syringes stolen. The police take them…” Adverse police behaviors in rural communities also manifested on social media via law enforcement officials implicating SSPs as responsible for syringe possession and litter. For instance, one participant explained that the Sherriff’s Department would take pictures of syringes, post them on social media, and tag the health department: “For a short time, our sheriff’s department was tagging the health department every time they pulled someone over and seized needles. They would take pictures of them and tag us on Facebook.”

### Engaging with rural law enforcement

Many participants described the importance of rural SSP advocates cultivating relationships with members of law enforcement to facilitate meaningful conversations about SSPs and dispel concerns persons may have about program operations. For example, a participant explained that rural SSP proponents should engage law enforcement in discussions about SSPs prior to publicly announcing their intent to launch programs and provide persons with opportunities to ask questions:

The advice I give to all my public health friends is do not let the Police Chief hear about this (SSP implementation) in the newspaper, on the radio, or in the City Council meeting. Go sit down with him or her first. ‘Here’s what we’re thinking about asking for. What are your thoughts on it? Here’s why we think it will work.’ Same thing with the local sheriff.

Participants also explained that communicating with rural law enforcement about SSPs prior to program implementation provided an opportunity to educate persons about the potential influence of policing practices on the public health of PWID; for example, a participant explained:

It was more like asking law enforcement not to give people a hard time–like setting up, looking for people going there, try not to park around the Health Department on days that it’s happening. We don’t want people to be scared off. It was more like, ‘Help us help the community,’ and they (law enforcement) seemed to be fine with it.

### Needlestick prevention messaging

In describing how participants acquired support from law enforcement for rural SSP implementation, they routinely emphasized the importance of communicating how police officers may personally benefit from SSPs. In particular, participants described the critical role of explaining how SSP implementation may carry protective effects against needlestick injuries. As succinctly stated by a participant:

…because really what those guys (law enforcement) want to hear is, ‘What is it (SSP implementation) gonna’ do for me?’ Right? They're not so much concerned about the drug user's health or anything like that, but is it gonna’ prevent needlesticks?

Similarly, a participant described how they incorporated information about needlestick injuries into their presentations to law enforcement about SSPs, “…and so part of the presentations that I would make would point out that police officers do benefit from these programs and that [their implementation] would reduce the likelihood of a needlestick happening.”

## Discussion

The results of this study suggest that there are multiple ways in which SSP implementation and subsequent operations in rural Kentucky counties are affected by law enforcement. Participants described scenarios in which law enforcement in rural communities advocated for SSP implementation; however, other participants reported police opposing SSP implementation and engaging in adverse behaviors that may jeopardize the public health of PWID. Advocates for rural SSP implementation emphasized the importance of communicating with law enforcement about SSPs and working to resolve their concerns. Notably, participants reported that efforts to educate law enforcement about the necessity for SSPs were particularly impactful when they discussed how SSP implementation may prevent needlestick injuries. Our findings fill a gap in the literature given that few studies have explored the relationship between SSP implementation and law enforcement in rural areas.

Participants described having to confront inaccurate and stigmatizing beliefs held by law enforcement officers in rural communities about SSP operations (e.g., programs enabled drug use). Extensive scientific literature documents the public health benefits of SSP implementation and dispels myths about their community-level impacts [1–4, 7, 28]. For example, SSP implementation has not been shown to enable or encourage drug use, nor do SSPs lead to increased syringe litter or crime [1, 10–12, 29, 30]. With more than thirty years of scientific evidence that supports SSP implementation, renewed efforts are warranted to develop strategies that ensure key constituency groups in rural communities, including law enforcement, understand the evidence about SSPs [31]. Innovations in public health messaging are a high priority given that rural law enforcement is routinely positioned to affect initial SSP implementation and subsequent operations.

Efforts to cultivate support for SSP implementation among law enforcement in rural communities may benefit from educating persons about how SSP implementation may reduce needlestick injuries [32]. Participants in our study emphasized that while it was important to frame rural SSP implementation through a public health lens, tailoring messaging such that it focused on how SSPs may benefit law enforcement personally (i.e., via reducing needlestick injuries) was an advantageous strategy. This finding has many parallels to emerging research that examines how law enforcement officials may interact with SSPs and their clients. For example, a recent study found that police officers who endorsed SSPs were less likely to believe that SSP implementation increased risks for needlestick injury [33]. In addition, a 2019 study found that law enforcement officials who experienced a needlestick injury were more critical of SSPs, reporting that they believed SSPs enabled drug use [17]. Another study found that police officers who had experienced a needlestick injury had greater odds of syringe confiscation [34]. Future work is needed to better understand how to address the concerns held by members of law enforcement in rural communities concerning needlestick injuries while bolstering support for SSP implementation and sterile syringe access among PWID.

Similar to existing urban-based research, participants in our study reported that law enforcement in rural communities engaged in policing behaviors that may adversely affect SSP operations and the health of PWID [14–16, 20]. For instance, police parked near county lines to target PWID returning from SSPs in adjacent counties. In other instances, participants described policing behaviors in which rural law enforcement targeted PWID who accessed SSPs, charged them with drug possession, and confiscated their sterile injection supplies. These adverse policing behaviors carry substantial implications for the prevention of infectious disease transmission among PWID populations [15, 21, 35–37]. In addition, intensified police presence and targeting PWID may lead to unsafe injection practices (e.g., rushing injection, discourage safer injection practices), increasing risks for both overdose and infectious disease acquisition [19]. Given the number of rural counties that are vulnerable to injection drug use-associated infectious disease outbreaks and worsening trends in the overdose crisis, immediate actions should be taken to protect rural PWID from unnecessary policing behaviors that jeopardize public health [24].

Participants in our study described scenarios in which members of rural law enforcement adversely affected SSP implementation; however, they also reported instances of law enforcement supporting, and even championing, SSPs. As noted by our participants, response to rural SSP implementation among law enforcement varied considerably, from total opposition to vocal support. The heterogeneous response to SSP implementation among rural law enforcement warrants additional research. In particular, future studies should aim to identify and explore the combinations of underlying factors that collectively influence how rural law enforcement perceives SSP implementation. Participants also elaborated that law enforcement advocating for SSP implementation in rural communities carried significant weight at the local level during processes to acquire approvals for program operations. Future work should be conducted to better understand how rural communities can work together in ways that reflect evidence-based practices and support the health of vulnerable populations, including PWID.

This research is not without limitations. Our findings only reflect the perspectives of persons who were involved in SSP implementation and subsequent operations in rural counties in Kentucky. The layered approval process for SSP implementation in Kentucky may have affected the degree to which persons interacted with rural law enforcement and how they subsequently described their relationship with SSP implementation. Our findings should be interpreted within this context, as SSP approval and implementation policies vary. Importantly, we did not interview active members of the law enforcement community to explore their perspective on SSP implementation. Future work should be conducted to comprehensively explore the attitudes and beliefs law enforcement officials in rural communities have about SSP implementation. Recall bias is another potential limitation of this research given that some SSPs had been operational for several years prior to our interviews. Despite these limitations, this study fills an important gap in the scientific literature by shedding light on how law enforcement affected SSP implementation and subsequent operations in rural counties in Kentucky.

## Conclusion

In conclusion, this research demonstrates that law enforcement may play a prominent role during SSP implementation processes in rural counties, ranging from vocal support to engaging in policing behaviors that may adversely affect the public health of PWID. Law enforcement can be a powerful champion for SSP implementation but may require extensive and sustained outreach and education efforts to ensure their attitudes and beliefs reflect empirical research evidence. Engaging rural law enforcement officials in discussions about SSP implementation may be particularly impactful via incorporating messaging about how SSPs may decrease risks for needlestick injuries. Future work is needed to explore how to expeditiously engage rural law enforcement, and communities more broadly, about SSPs, their benefits, and public health necessity.

## Data Availability

The datasets generated and/or analyzed during the current study are not publicly available in order to protect participant confidentiality.

## Abbreviations

SSP: Syringe services programs
PWID: People who inject drugs
CFIR: Consolidated Framework for Implementation Research
STIs: Sexually transmitted infections
HIV: Human immunodeficiency virus
